# Neuroprotective mechanisms of Buyang Huanwu decoction in ischemic stroke

**DOI:** 10.3389/fphar.2025.1620533

**Published:** 2025-09-23

**Authors:** Yuanyuan Qin, Shiliang Hu, Shiman Mawen, Shanyao Pan, Yaping Huai, Guoqiang Liang, Ting Chen, Feiyan Zhao, Hongli Dong, Xuyi Yao, Xue Wu, Zhigang Lv, Jiao Deng, Fei Huang, Li Luo

**Affiliations:** ^1^ Suzhou TCM Hospital Affiliated to Nanjing University of Chinese Medicine, Suzhou, China; ^2^ School of Physical Education and Sports Science, Soochow University, Suzhou, China; ^3^ Department of Rehabilitation Medicine, Shenzhen Longhua District Central Hospital, Shenzhen, China; ^4^ Department of Rehabilitation Medicine, Changzhou Hospital of Traditional Chinese Medicine, Changzhou, China

**Keywords:** ischemic stroke, Buyang Huanwu decoction, neuroinflammation, oxidative stress, neuroprotection

## Abstract

Ischemic stroke (IS) continues to be a major contributor to global mortality and long - term disability. Buyang Huanwu Decoction (BHD), a traditional Chinese medicine formula, has shown effectiveness in reducing brain injury and promoting post - stroke recovery through experimental researches and clinical trials. The neuroprotective mechanisms of BHD against cerebral ischemic injury involve multiple pathways, such as suppression of inflammation, reduction of oxidative stress, inhibition of apoptosis, regulation of autophagy, and enhancement of mitochondrial function. Moreover, BHD presents therapeutic potential by boosting neuroplasticity, enhancing angiogenesis, reducing excitotoxicity, optimizing brain energy metabolism, and regulating gut microbiota. Considering the current scarce effective treatments for IS, exploring BHD’s therapeutic potential and its mechanism holds substantial clinical significance. This review systematically organizes recent research advancements on BHD’s application in IS management and its underlying mechanisms, providing useful insights for future research and clinical practice.

## 1 Introduction

Stroke is a leading cause of death and long-term disability worldwide, owing to its high incidence and devastating sequelae (GBD 2016 Stroke Collaborators, 2019). IS, which is most often due to thrombotic vessel occlusion, comprises the majority of stroke cases and results in cerebral ischemia and hypoxia (Campbell et al., 2019). Current treatments—thrombolysis, antiplatelet therapy, and neuroprotective agents—face well-known limitations: a narrow therapeutic window, patient ineligibility or drug insensitivity, and significant post-treatment complications (Cheng et al., 2024; Yang et al., 2025). More than two-thirds of stroke survivors sustain persistent neurological deficits—manifesting as motor, cognitive (including language), sensory, and cardiopulmonary impairments (Crichton et al., 2016; Benjamin et al., 2018). Conventional therapeutic interventions, including pharmacotherapy, rehabilitation therapy, and secondary prevention, have shown very limited efficacy (Tg et al., 2020). Consequently, there is an urgent need to identify more effective therapeutic strategies.

Traditional Chinese Medicine (TCM) has been widely used as an adjunctive therapy for post-stroke sequelae in China, featuring multi-target effects and low side effects (Hu et al., 2018; Zhang W.-W. et al., 2018). Studies have demonstrated that combining TCM treatment with conventional therapies can improve neurological symptoms and activities of daily living in stroke patients (Cai et al., 2019; Gao et al., 2021). BHD, a classic TCM formula, was first recorded in *Yilin Gaicuo* (*Corrections of Errors in Medical Works*) by Wang Qingren in the Qing Dynasty, and is used for treating post-stroke sequelae due to qi deficiency and blood stasis syndrome. The formula consists of seven ingredients: *Astragalus membranaceus (Huangqi), Angelica sinensis (Danggui), Paeonia lactiflora var. chinensis (Chishao), Lumbricus (Dilong), Persicae Semen (Taoren), Carthami Flos (Honghua), and Ligusticum chuanxiong (Chuanxiong)* in a ratio of 120:6:4.5:3:3:3:3. BHD is widely used in clinical practice to promote the recovery of neurological and motor functions, benefiting patients with post-stroke sequelae, with no reported adverse reactions (Gao et al., 2021; Shao et al., 2022; Wang et al., 2022). In addition, in experimental stroke models, BHD can reduce cerebral infarct volume, improve neurological prognosis, and inhibit oxidative stress and neuronal apoptosis (Cai et al., 2007; She et al., 2023; Chen et al., 2024). However, the specific mechanisms underlying the role of BHD in stroke recovery remain incompletely elucidated.

This formula contains several bioactive components, including astragaloside IV and isoflavonoids from *Astragalus membranaceus*, paeoniflorin from *Paeonia lactiflora*, Hydroxy-safflor yellow A from *Carthami Flos*, and ligustrazine from *Ligusticum chuanxiong*. Studies have shown that these components exert multiple neuroprotective effects, such as promoting neurogenesis, inhibiting oxidative stress and inflammation, preventing thrombosis, protecting the blood-brain barrier, and modulating apoptosis following cerebral ischemia (Fu et al., 2014; Jiang et al., 2020; Wu et al., 2020; Wang et al., 2021; 2025). BHD, as an organic combination based on TCM theory, exhibits multi-component, multi-pathway, and multi-target effects. The interactions between its components may involve synergistic, antagonistic, or sensitizing effects. Numerous studies have demonstrated that BHD has a certain degree of neuroprotective effect in ischemic stroke, and its mechanisms are complex and diverse. The therapeutic efficacy results from the combined action of its ingredients. For example, *Ligusticum chuanxiong*, a key “guide” herb, increases the distribution of other ingredients in the brain; *Astragalus membranaceus* slows down the metabolism of paeoniflorin, maintaining its activity; and ligustrazine enhances the transmembrane transport of paeoniflorin, highlighting the scientific and rational compatibility of this formula (Zheng et al., 2018; Liu et al., 2021). Although the research on the individual active components provides important insights into the pharmacological basis of BHD’s therapeutic effects, the essence of TCM formulas lies in their “holistic view.” A TCM formula is an organic whole formulated under the guidance of TCM theory, and its efficacy arises from the combined effects of multiple components, pathways, and targets. The components may exhibit complex interactions, such as synergy, antagonism, or sensitization, rather than a simple additive effect of individual components. Therefore, this study will focus on the overall effects of the entire BHD formula, rather than isolating the targets of single components. It aims to systematically summarize the network pharmacology map of BHD’s multi-mechanistic, synergistic treatment of stroke, providing valuable references for its clinical application and offering direction for future research.

## 2 The mechanisms of BHD in the treatment of ischemic stroke

**FIGURE 1:**
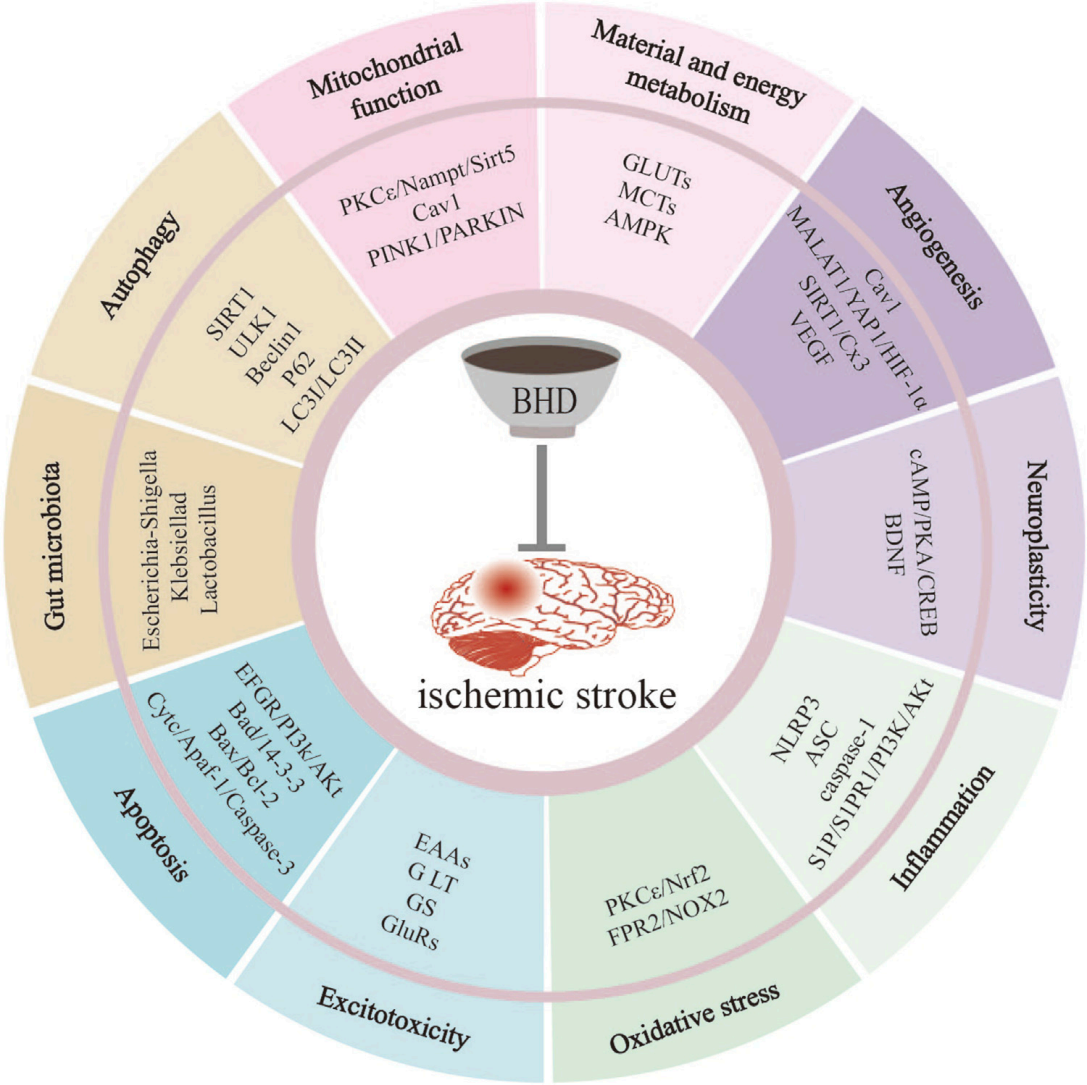
BHD mitigates IS-induced injury through multiple pathways. These pathways include suppression of inflammation, reduction of oxidative stress, inhibition of apoptosis, regulation of autophagy, improvement of mitochondrial function, promotion of neuroplasticity, promotion of angiogenesis, inhibition of excitotoxicity, regulation of material and energy metabolism, regulation of gut microbiota.

Extensive preclinical studies demonstrate that BHD effectively attenuates cerebral ischemia-reperfusion (I/R) injury. In this review, we synthesize these findings to elucidate BHD’s molecular mechanisms—focusing on the principal pathways and targets that underlie its neuroprotective actions (Figure 1).

### 2.1 Suppression of inflammation

Neuroinflammation is a critical target for mitigating post-stroke damage and promoting recovery (Iadecola and Anrather, 2011; Kl et al., 2019). Neuronal necrosis following ischemic stroke releases damage-associated molecular patterns (DAMPs) and pro-inflammatory mediators, which activate microglia and astrocytes and recruit peripheral immune cells into the cerebral ischemic penumbra (Shi et al., 2019). Activated glia and infiltrating leukocytes then amplify local inflammation *via* overproduction of cytokines—a response tightly connected to systemic immune alterations (Iadecola et al., 2020; Simats and Liesz, 2022).

**FIGURE 2:**
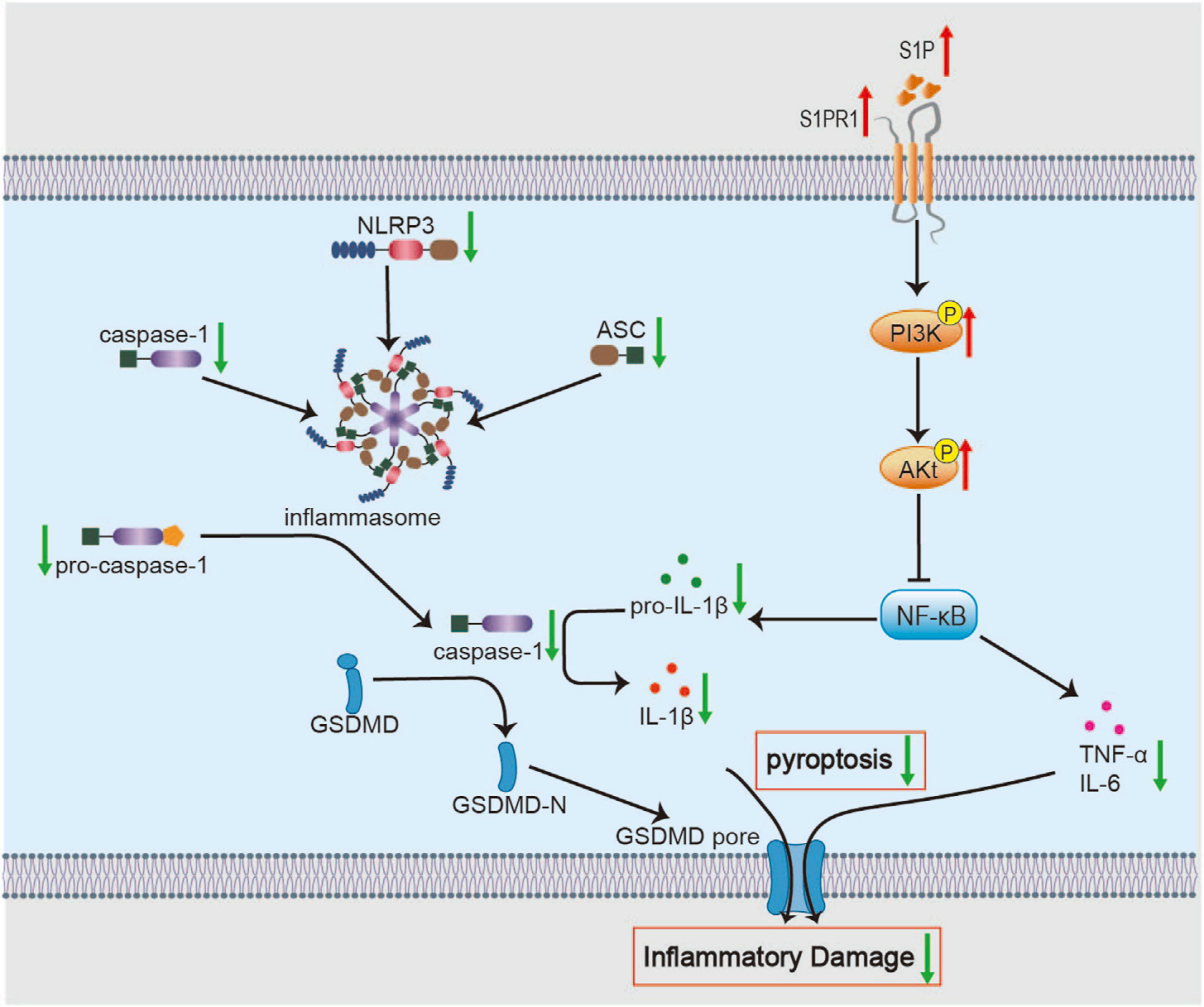
Molecular mechanisms of BHD in suppressing neuroinflammation. BHD alleviates neuroinflammation by inhibiting the NF-κB signaling pathway and its downstream molecules *via* activation of the S1P/S1PR1/PI3K/Akt axis. Concurrently, this inhibition suppresses NLRP3 inflammasome assembly and the activity of downstream pyroptosis effectors, thereby reducing the secretion of pro-inflammatory cytokines and attenuating inflammatory tissue damage.

Importantly, pyroptosis—a caspase-1-dependent form of inflammatory cell death—has emerged as a major driver of ischemic stroke pathology, primarily through activation of the canonical Nucleotide-binding domain and leucine-rich repeat-containing pyrin domain 3 (NLRP3) inflammasome (Adamczak et al., 2014; Tan et al., 2014). A growing body of evidence indicates that NLRP3 inflammasome activation markedly amplifies neuroinflammation and exacerbates I/R injury (Li J. et al., 2023). Studies have shown that pre-treatment with 7 days of BHD significantly enhances the brain’s tolerance to subsequent ischemia/reperfusion damage, as evidenced by a reduction in infarct volume and an improvement in neurological function scores 24 h post-reperfusion. This pharmacological preconditioning effect is likely associated with the downregulation of key NLRP3 inflammasome components (ASC, pro-caspase-1) and pyroptosis effectors (active caspase-1, IL-1β) (Figure 2) (She et al., 2019). Notably, astragaloside IV and Hydroxysafflor Yellow A may be key active ingredients of BHD in suppressing pyroptosis (Hou et al., 2024). Since NLRP3 inflammasome components are expressed across multiple cell types in the ischemic brain and drive pyroptosis (Fann et al., 2014; Jorgensen and Miao, 2015), targeting NLRP3-mediated inflammation presents a promising avenue for therapeutic intervention in ischemic stroke. Future research should explore the potential application of BHD’s preconditioning advantage in clinical high-risk populations.

Notably, the role of neuroinflammation—and BHD’s modulation of it—is stage-dependent. In the acute phase, BHD primarily suppresses deleterious, excessive inflammation to mitigate secondary injury. As the disease advances into the recovery phase, the inflammatory response assumes a more complex, dual role. Studies show that BHD promotes polarization of microglia toward an M2 phenotype and astrocytes toward an A2 phenotype in middle cerebral artery occlusion (MCAO)/R rats during recovery, thereby facilitating synaptogenesis and neurite outgrowth (Li et al., 2024c). Moreover, Liu W demonstrated that in the permanent MCAO (pMCAO) mouse model, BHD treatment consistently promoted long-term neurological recovery, with improvements in neurological deficits and reduced infarct volume observed on days 7 and 14 post-stroke. The recovery benefits were closely related to the activation of the Sphingosine-1-Phosphate (S1P)/Sphingosine-1-Phosphate Receptor 1 (S1PR1)/Phosphatidylinositol 3-Kinase (PI3K)/Protein Kinase B (PKB, Akt) survival and repair signaling pathway (Liu W. et al., 2023). The PI3K/Akt/nuclear factor kappa B (NF-κB) signaling cascade is a core regulator of post-ischemic neuroinflammation (Li L. et al., 2023; Li J. et al., 2024). Upstream, S1P activates S1PR1 to promote Akt phosphorylation, thereby exerting neuroprotective effects in ischemic models (Hasegawa et al., 2010). As a bioactive sphingolipid, S1P/S1PR1 signaling mitigates inflammatory injury and supports neural repair (Nakamura et al., 2021; Zaibaq et al., 2022). These results implicate S1P/S1PR1 as a potential direct target of BHD. Moreover, most evidence derives from whole-brain homogenates in rodent models. Future work should validate these mechanisms in isolated cell populations—such as microglia and neurons—to delineate cell-type–specific effects of BHD.

In addition to local inflammation, ischemic stroke induces systemic immunosuppression, which profoundly affects recovery. Initially, DAMPs and cytokines leak into the circulation *via* a disrupted blood-brain barrier, provoking transient systemic immune activation. This phase swiftly gives way to sustained immunosuppression, heightening the risk of complications such as stroke-associated pneumonia (Iadecola et al., 2020; Wang et al., 2023). Concomitant splenic atrophy and lymphocyte apoptosis further exacerbate secondary neural damage (Yu H. et al., 2021). Fu R found that BHD reduces splenic T-cell apoptosis at 3 days post-MCAO/R, ameliorating both cerebral injury and systemic immunosuppression—possibly *via* the Absent in melanoma 2 (AIM2)/IL-1β/Fas ligand-Fas receptor (FasL-Fas) axis. Moreover, quercetin from safflower may contribute to this process by inhibiting peripheral immune cell recruitment (Zhang et al., 2022). Yet, direct evidence for AIM2 dependence is lacking (Fu et al., 2024). However, it remains necessary to verify whether BHD exerts this effect specifically *via* AIM2. Notably, Roth S reported that AIM2 inhibition did not alter neurological outcomes within 24 h post-stroke, suggesting that timing critically influences AIM2’s role (Roth et al., 2021). Accordingly, future studies should dissect the temporal and spatial dynamics of BHD’s effects on splenic immune subsets and map the communication pathways of key immune mediators between brain and spleen.

### 2.2 Reduction of oxidative stress

ATP depletion after ischemia leads to mitochondrial dysfunction and overproduction of reactive oxygen species (ROS). The resulting increase in malondialdehyde (MDA) and decrease in superoxide dismutase (SOD) activity exacerbate oxidative injury, damaging organelles and compromising neuronal viability (Liu et al., 2018; Yang et al., 2018; Kamal et al., 2023).

**FIGURE 3:**
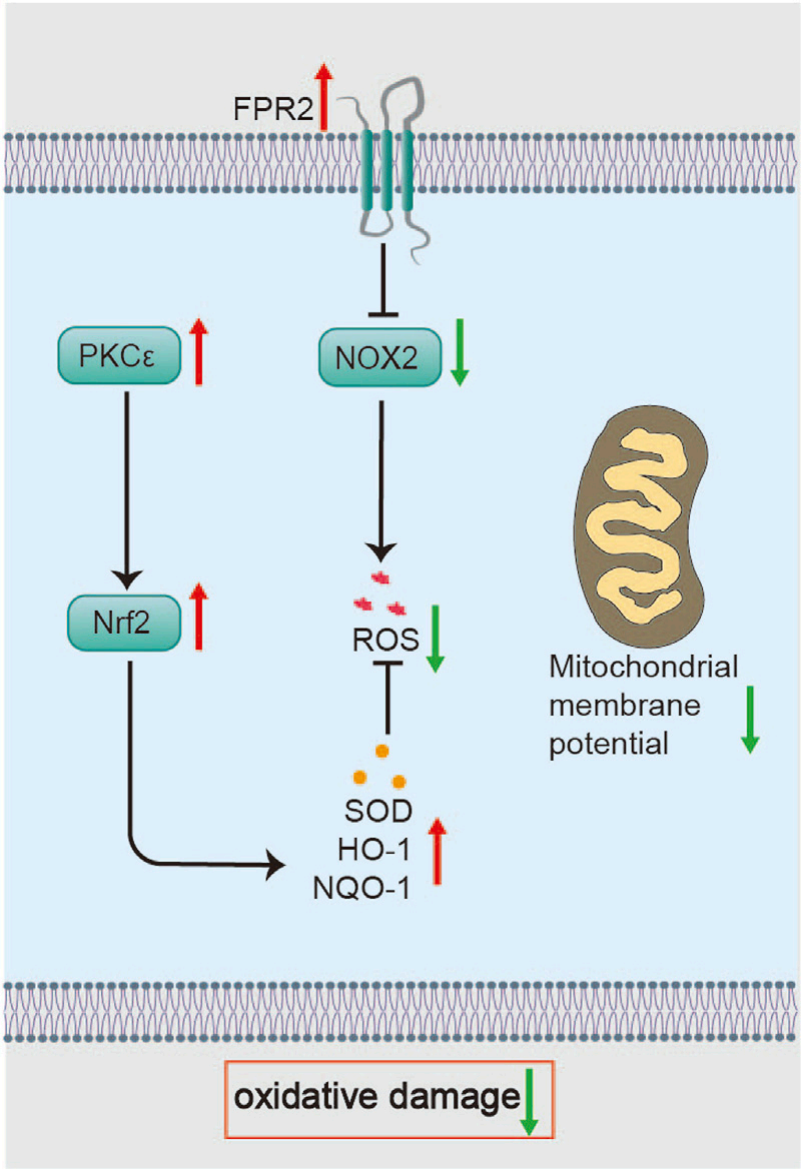
Molecular mechanisms of BHD in restoring mitochondrial function. BHD regulates mitochondrial dynamics *via* the PKCε/Nampt/Sirt5 axis and restores mitochondrial function, potentially involving Cav-1-mediated effects on MQC processes.

Li C showed that BHD scavenges ROS in isolated cerebral cells from MCAO/R rats and preserves neuronal membrane fluidity (Li, 2012). In in vivo experiments, BHD enhances the antioxidant defense capability in MCAO/R rats on day 3, restores mitochondrial membrane potential, reduces neuronal death, and decreases infarct size. Mechanistically, BHD upregulates protein kinase C epsilon (protein kinase Cε, PKCε), promoting nuclear factor erythroid 2-related factor 2 (Nrf2) nuclear translocation and the subsequent induction of antioxidant enzymes, including SOD, heme oxygenase-1 (HO-1), and NAD(P)H quinone dehydrogenase 1 (NQO1) (Yin et al., 2023). Nrf2, the master regulator of antioxidant defense, maintains redox balance by driving both basal and inducible expression of enzymes that neutralize ROS and electrophiles (Figure 3) (Zhang et al., 2021). Notably, compared to edaravone—an ROS scavenger that acts *via* direct chemical quenching—BHD uniquely restores endogenous antioxidant capacity through enzyme induction (Dickmeiß et al., 2025; Lee et al., 2025). This highlights BHD’s antioxidative stress effect during the acute phase of cerebral ischemia. This effect may be mediated by astragaloside IV and Quercetin through the activation of the Nrf2 antioxidant signaling pathway (Li et al., 2018; Zhang et al., 2022). Future work should identify the intermediate signaling factors that link BHD to PKCε activation and investigate Nrf2-independent mechanisms of mitochondrial protection.

Additionally, Wu F proposed that BHD might exert its antioxidative effects in the acute phase of ischemic stroke through the Formyl peptide receptor 2 (FPR2)/NADPH oxidase 2 (NOX2) signaling pathway (Wu et al., 2021). FPR2—a neuroprotective GPCR abundant in the brain—when deficient, worsens I/R injury (Sa et al., 2016; Bisicchia et al., 2018). Since NOX2 is a primary source of ROS, its inhibition dampens oxidative bursts (Figure 3) (Khayrullina et al., 2015). Therefore, BHD is an effective remedy for combating oxidative stress in the acute phase. Nonetheless, it remains unclear whether BHD directly modulates NOX2 activity or acts upstream *via* FPR2.

### 2.3 Inhibition of apoptosis

Cerebral I/R activates complex apoptotic cascades, which are central to neuronal loss and ensuing neurological deficits. These cascades consist of the intrinsic (mitochondria-mediated) pathway—driven by mitochondrial outer-membrane permeabilization and calpain activation—and the extrinsic pathway, triggered by death receptors in response to cytokines and chemokines (Tuo et al., 2022). In the intrinsic pathway, injury-induced mitochondrial outer membrane permeabilization (MOMP) releases cytochrome c into the cytosol. Cytochrome c then associates with apoptotic protease-activating factor 1 (Apaf-1) to form the apoptosome, which initiates the caspase cascade and orchestrates programmed cellular disassembly (Glover et al., 2024). Members of the B-cell lymphoma 2 (Bcl-2) family tightly regulate MOMP: anti-apoptotic Bcl-2 prevents cytochrome c release, whereas pro-apoptotic Bcl-2-associated X protein (Bax) facilitates membrane permeabilization (Shore and Nguyen, 2008; Soriano and Scorrano, 2011).

Liu F reported that BHD suppresses Cyclin-dependent kinase 5 (CDK5) and Tau overexpression in H_2_O_2_-stressed neuronal cells, concomitantly downregulating caspase-3 activity and reducing the Bax/Bcl-2 ratio (Liu et al., 2019). CDK5 can trigger apoptosis by phosphorylating Bcl-2 family members at the mitochondrial membrane or directly modifying executioner caspases such as caspase-3 and caspase-7 (García-Sáez, 2012; Maitra and Vincent, 2022). Nevertheless, Liu et al. did not confirm a causal link between CDK5 inhibition and downstream apoptotic markers, underscoring the need for *in vivo* validation. In a separate study, Song C demonstrated that serum from BHD-treated MCAO/R rats protects Oxygen-Glucose Deprivation/Reperfusion (OGD/R)-injured brain microvascular endothelial cells—enhancing viability, reducing TUNEL positivity, lowering Bax and caspase-3 levels, and increasing Bcl-2. They further showed that BHD suppresses glycolysis-driven histone H3 lactylation to downregulate Apaf-1 transcription (Song et al., 2024). However, the multifaceted composition of medicated serum raises the possibility of confounding by non-BHD factors. Notably, Paeoniflorin and Amygdalin may be key active components of BHD in mediating its anti-apoptotic effects (Zhang Y. et al., 2015; Kimura et al., 2025).

**FIGURE 4:**
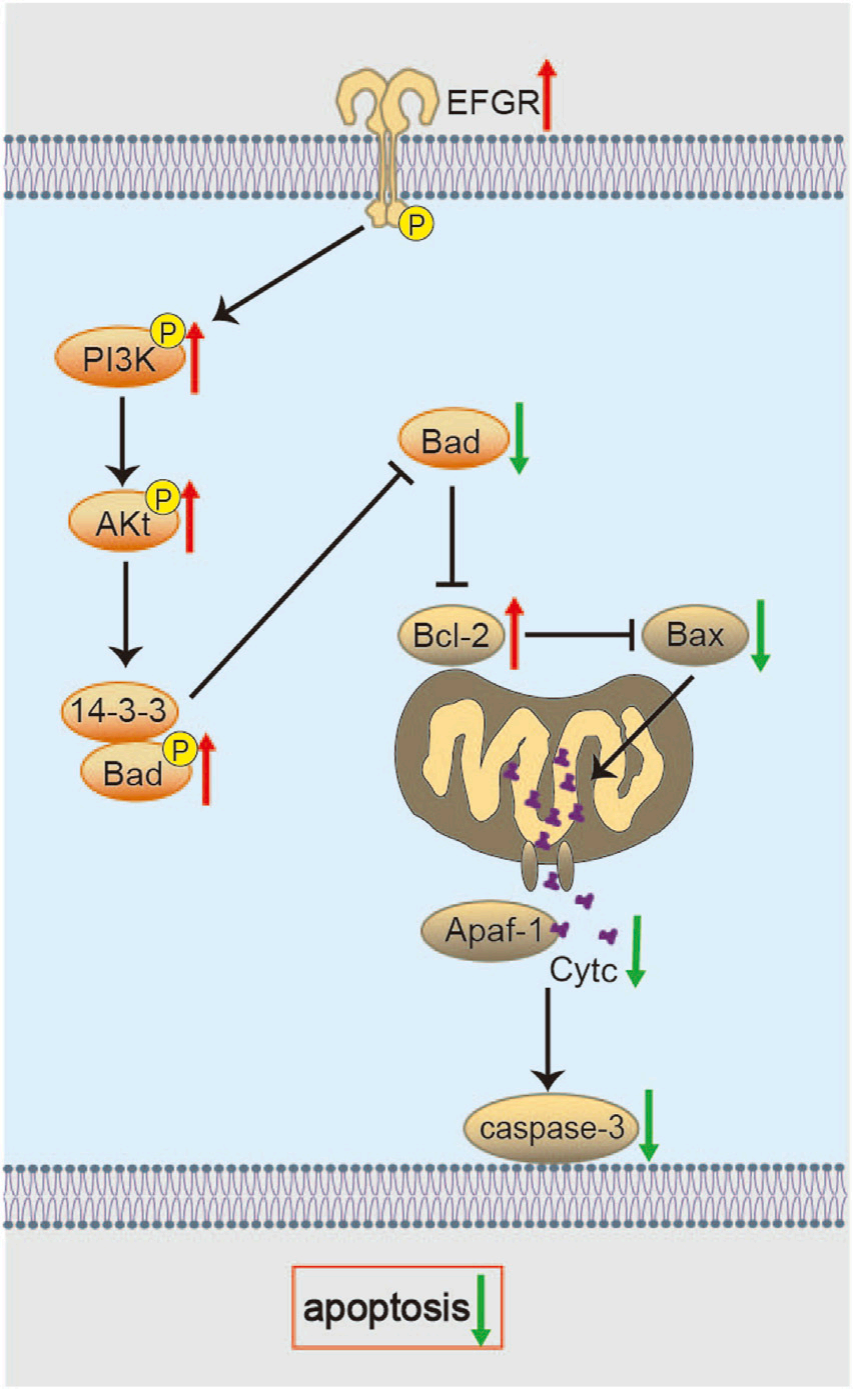
Molecular mechanisms of BHD in suppressing oxidative stress. BHD restores mitochondrial membrane potential and enhances antioxidant enzymes *via* the PKCε/Nrf2 axis, while potentially reducing ROS generation through FPR2/NOX2 signaling to alleviate oxidative damage.

Chen et al. used proteomic analysis to find that, after 14 days of BHD intervention in the MCAO/R model, BHD significantly alleviated neuronal apoptosis. Mechanistic studies suggest that this effect might be mediated through the activation of the epidermal growth factor receptor (EGFR)/PI3K/Akt signaling axis, which then regulates downstream Bcl-2-associated death promoter (Bad) and 14-3-3 protein signaling (Figure 4) (Chen et al., 2020). In this paradigm, Akt-mediated phosphorylation of Bad fosters its sequestration by 14-3-3 proteins, thereby blocking Bax activation, cytochrome c release, and caspase-3 induction (Datta et al., 2000; Nomura et al., 2015). Therefore, BHD may directly enhance the intrinsic pro-survival signaling network in the recovery phase after cerebral ischemia, providing a stable cellular environment for neuronal repair. However, the cell type-specificity of this signaling pathway (such as its effect on neurons, astrocytes, or oligodendrocytes) and the indispensability of each signaling node (e.g., EGFR, PI3K) in mediating BHD’s effects still require experimental validation using cell-specific knockout models.

### 2.4 Regulation of autophagy

Autophagy can protect neurons during cerebral ischemia by removing damaged organelles and misfolded proteins (Dugbartey, 2024; Newton et al., 2024), and it remains crucial for restoring cellular homeostasis during reperfusion (Liu S. et al., 2023). Nevertheless, the protective role of autophagy in I/R injury remains context-dependent (Aghaei et al., 2019; Yang et al., 2019), as excessive or prolonged dysregulation can be detrimental (Gao et al., 2012; Sun et al., 2018). Therefore, precise temporal regulation of autophagy is required at each post-ischemic stage. Studies have shown that after 2 h of ischemia and 3 days of reperfusion, the levels of Microtubule-associated protein 1 light chain 3 (LC3) II/I and Beclin 1 autophagy related gene (Beclin-1) in the ischemic penumbra of MCAO/R rats were significantly elevated (Shu et al., 2016; Pan et al., 2020). However, in a 1.5-h ischemia MCAO/R model, the levels of Beclin-1 and LC3 II in the ischemic penumbra were significantly reduced at 24 h and 7 days post-surgery (Wu et al., 2018). These differences might be attributed to variations in ischemia and reperfusion times in the models.

Zhao Y found that BHD reduced Beclin-1 and LC3-II levels in the ischemic penumbra at day 3 post-MCAO/R, with no changes in the ischemic core or contralateral hemisphere. However, assessing only Beclin-1 and LC3-II risks conflating reduced autophagosome formation with impaired autophagic flux (Zhao et al., 2021). In contrast, Li H reported that by day 5 post-reperfusion, BHD not only reduced infarct size but also elevated Beclin-1 and LC3-II, decreased Sequestosome 1 (p62), and upregulated Sirtuin 1 (SIRT1) in the penumbra (Li et al., 2021). Given that SIRT1 directly deacetylates autophagy regulators such as Beclin-1 and Unc-51 like autophagy activating kinase 1 (ULK1) complex components (Thapa et al., 2024), these findings suggest a SIRT1-dependent mechanism—though direct evidence for SIRT1’s necessity in BHD-induced autophagy remains lacking. Future work should employ SIRT1 loss-of-function models (e.g., genetic deletion or pharmacological inhibition) to verify its role in BHD-induced autophagy and neurogenesis, and use co-immunoprecipitation or proximity assays to confirm direct SIRT1–Beclin-1/ULK1 interactions. Because autophagy dynamics evolve over time, comprehensive flux mapping at days 1, 3, 5, and 7 post-ischemia—using metrics such as p62 degradation rates, LC3-II puncta quantification, and mRFP-GFP-LC3 reporter assays—is essential for delineating BHD’s temporal effects. Qin B also demonstrated that BHD enhances autophagy in OGD/R-injured neural stem cells—upregulating Beclin-1 and LC3-II while reducing p62 (Qin et al., 2021). However, without full flux measurements or identification of upstream receptors and signaling intermediates, the mechanistic basis remains unclear. Integrating transcriptomic, proteomic, and metabolomic analyses in both *in vitro* and *in vivo* models will be crucial to pinpoint the precise molecular targets of BHD in autophagy regulation.

Overall, a principal function of BHD may be to restore autophagic homeostasis: it can attenuate excessive autophagic flux in the acute phase to prevent autophagy-dependent cell death, while in the subacute phase it can promote basal autophagy to facilitate clearance of damaged organelles and proteins, thereby supporting cellular repair and survival. This dynamic adaptation to the evolving post-stroke pathological milieu may be a key advantage of multi-herb formulas such as BHD. Future studies using serial time-point analyses are essential to validate this temporally specific regulation and to define the optimal therapeutic window for BHD intervention.

### 2.5 Improvement of mitochondrial function

Mitochondrial quality control (MQC)—the suite of processes that preserve mitochondrial morphology, dynamics, and function—underlies organelle homeostasis and supports neuronal survival (Tian et al., 2022). Mitochondrial disruption during cerebral I/R has emerged as a key pathological driver that determines the extent of neuronal damage following stroke (Rutkai et al., 2019). Dysregulation of MQC mechanisms, including impaired mitophagy, altered fusion/fission balance, and defective biogenesis, exacerbates mitochondrial dysfunction and contributes to neuronal death following IS (Song et al., 2022; Tian et al., 2022). Restoring MQC has therefore emerged as a promising therapeutic strategy to mitigate secondary brain damage and enhance neurological recovery after IS (Yang et al., 2021).

**FIGURE 5:**
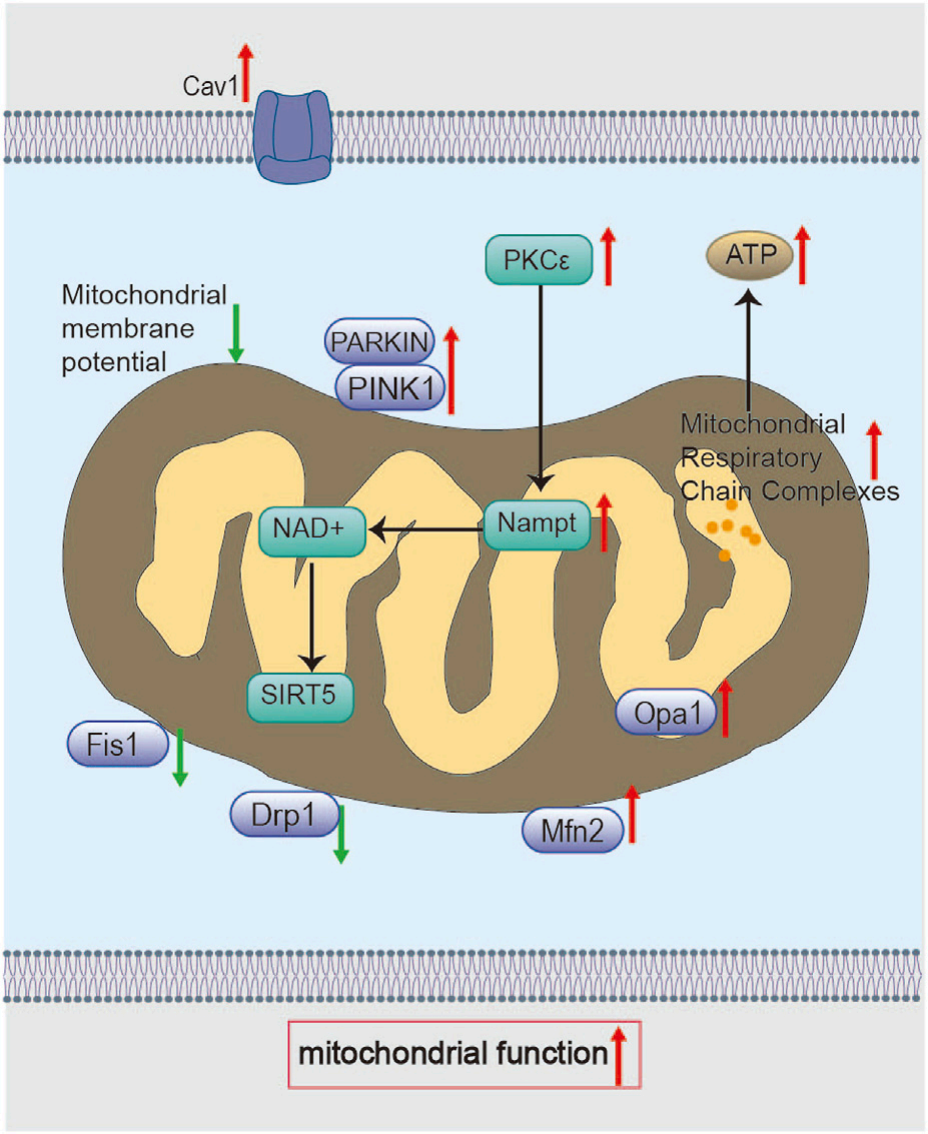
Molecular mechanisms of BHD in restoring mitochondrial function. BHD modulates the PKCε/Nampt signaling axis through Cav-1, elevates NAD^+^ levels, and activates Sirt5, which in turn upregulates the expression of mitochondrial fusion proteins (Mfn2, Opa1) and suppresses the expression of fission proteins (Drp1, Fis1), thereby regulating mitochondrial dynamics. In addition, BHD promotes mitophagy via the PINK1/Parkin pathway. Collectively, these mechanisms restore mitochondrial membrane potential, improve respiratory chain function, and enhance ATP production.

Studies demonstrate that BHD restores mitochondrial membrane potential and NAD^+^/NADH ratios, reduces infarct volume, and mitigates neuronal injury in MCAO/R model rats (Yin et al., 2024). Additionally, Liu Z found that, after 7 days of BHD treatment in MCAO/R rats, BHD regulated mitochondrial dynamics through the PKCε/nicotinamide phosphoribosyltransferase (Nampt)/Sirtuin 5 (Sirt5) signaling axis. By modulating the expression of mitochondrial fission proteins (Drp1, Fis1) and fusion proteins (Mfn2, Opa1), BHD restored mitochondrial function and alleviated ischemia-reperfusion injury (Figure 5) (Liu et al., 2025). Notably, Drp1-mediated mitochondrial fission might be activated by Ligustilide, a component of BHD (Wu et al., 2022). PKCε is a neuroprotective kinase that supports mitochondrial integrity. Downstream, Nampt elevates NAD^+^/NADH ratios and enhances neuronal survival after ischemia (Gomes et al., 2011; Morris-Blanco et al., 2016). Nampt’s elevation of NAD^+^ levels activates Sirt5 (Beaudoin et al., 2012), and Sirt5 overexpression in turn promotes mitochondrial fusion and limits organelle degradation (Polletta et al., 2015; Zou et al., 2018). However, the precise post-translational modifications through which BHD-induced Sirt5 activation alters fission/fusion machinery have not been defined. Studies in purified neuronal cultures are required to confirm these effects and rule out non-neuronal contributions.

Furthermore, Xu Y’s study reported that BHD preserved mitochondrial morphology, protected respiratory chain function (including complex activities, ATP content, and ATPase activity), regulated mitochondrial dynamics (Drp1, Fis1, Mfn2, Opa1), improved mitophagy (*via* the PINK1/Parkin pathway), and promoted mitochondrial biogenesis in MCAO/R rats 7 days post-intervention (Figure 5). They further demonstrated that Caveolin-1 (Cav-1) deficiency aggravates MQC disruption and diminishes BHD’s neuroprotection after ischemia (Xu et al., 2023). Cav-1 depletion likely impairs mitophagy and biogenesis, culminating in mitochondrial dysfunction (Bosch et al., 2011; Jiang et al., 2022). Cav-1 may facilitate the recruitment of fission/fusion proteins and mediate lipid trafficking within mitochondria (Xiao et al., 2022), but these mechanisms remain to be elucidated. Therefore, Cav-1 is a critical MQC regulator and a potential therapeutic target in ischemic stroke. Intriguingly, Chen X observed decreased Cav-1 levels after BHD treatment (Chen et al., 2020), a discrepancy that may arise from species differences, sampling timepoints, or brain regions analyzed.

These findings collectively suggest that the multi-targeted regulation of mitochondrial homeostasis is one of the key mechanisms through which BHD exerts its therapeutic effects during the recovery phase. Given the dynamic nature of mitochondrial remodeling, static measurements at a single timepoint are insufficient. Future investigations should leverage single-cell sequencing or spatial transcriptomics at multiple post-ischemic intervals to chart Cav-1’s spatiotemporal dynamics.

### 2.6 Promotion of neuroplasticity

Enhancing neuroplasticity is vital for functional recovery after ischemic stroke (Marques et al., 2019; Du et al., 2024). Early investigations showed that BHD stimulates proliferation and differentiation of neural stem cells in the cortex and dentate gyrus of MCAO/R rats (Sun et al., 2007; Gao et al., 2009). Additionally, BHD significantly improved neurological scores and preserved synaptic ultrastructural integrity in pMCAO rats, although it did not reduce infarct volume (Pan et al., 2017). However, electrophysiological studies are required to establish whether these structural improvements translate into enhanced neural circuit function.

**FIGURE 6:**
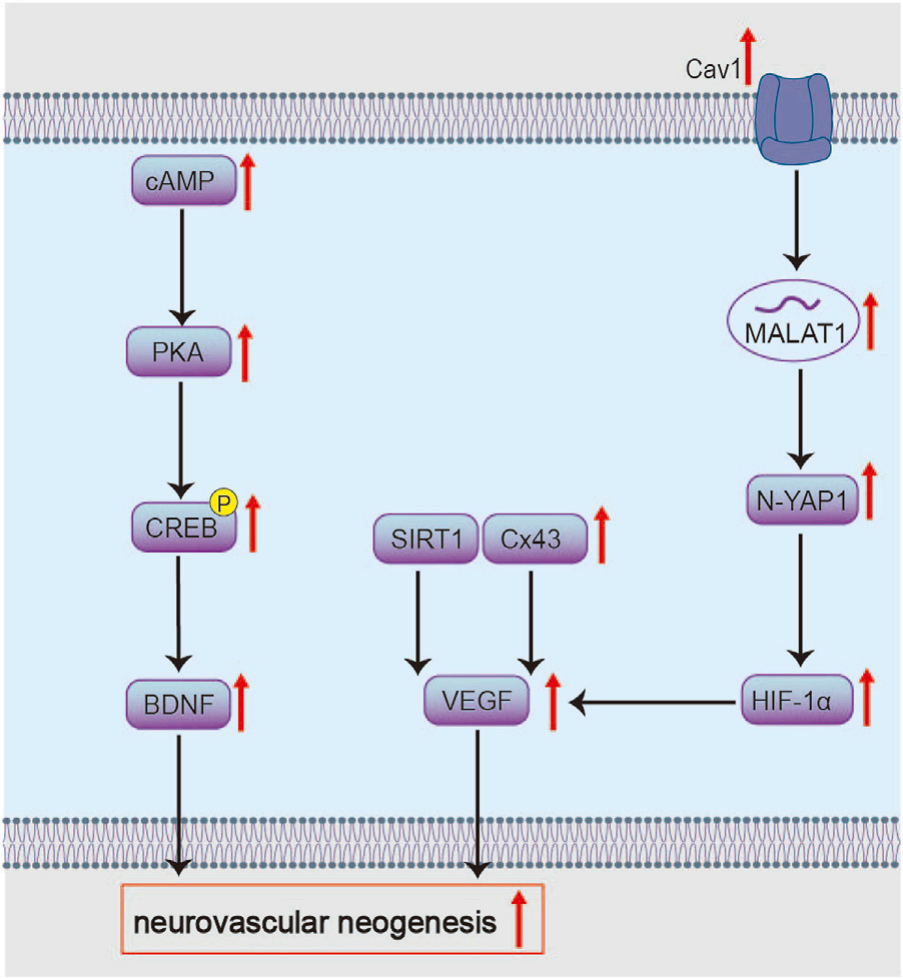
Molecular mechanisms of BHD in inhibiting apoptosis. BHD potentially inhibits Bad *via* the EGFR/PI3K/Akt/Bad/14-3-3 axis and modulates Bcl-2/Bax to control Cyt c release and caspase-3 activation, thereby suppressing apoptosis.

Li M et al. suggested that after 30 days of intervention in MCAO/R rats, BHD may promote neurite outgrowth and synaptogenesis *via* the AMP-activated Protein Kinase (AMPK)/cAMP Response Element-Binding Protein (CREB) pathway, a process associated with its ability to polarize microglia toward the M2 phenotype and astrocytes toward the A2 phenotype during stroke recovery (Li M. et al., 2024). This mechanism is supported at the compositional level: astragaloside IV, a key component of BHD, has been identified as an effective AMPK activator that drives M2 microglial polarization and facilitates axonal remodeling (Li et al., 2024c). Furthermore, after 7 days of intervention in MCAO/R rats, BHD ameliorated local pathology, increased dendritic spine density, and reduced neuronal apoptosis through the Cyclic Adenosine Monophosphate (cAMP)/Protein Kinase A (PKA)/CREB signaling axis (Figure 6) (Mo et al., 2024). Given that cAMP/PKA modulates growth, differentiation, metabolism, and cell survival (Khan et al., 2021). Activation of the PKA-CREB pathway positively influences learning and memory (Bae et al., 2019). Brain-derived neurotrophic factor (BDNF), a key CREB transcriptional target, promotes new synapse formation (Jiang et al., 2023). In summary, BHD synergistically activates CREB—a key transcription factor—through multiple signaling pathways during stroke recovery, thereby efficiently promoting neuroplasticity. Future studies should validate the crosstalk among these pathways at a cell-specific level and clarify which specific components in BHD initiate these upstream signals. Additionally, it is essential to identify the specific effector genes regulated by CREB that are influenced by BHD and to evaluate whether these structural changes enable new neurons to functionally integrate into existing neural networks.

Kong X reported that on days 7 and 21 after intervention in MCAO/R model rats, BHD may promote the proliferation, migration, and differentiation of neural progenitor cells (NPCs) by upregulating the expression of C-X-C Chemokine Receptor Type 4 (CXCR4) and Vascular Endothelial Growth Factor (VEGF) (Kong et al., 2014). However, direct evidence linking these factors to NPCs migration is lacking. Furthermore, VEGF’s dual role—in promoting angiogenesis and increasing blood-brain barrier permeability—raises concerns about potential exacerbation of edema (Zhang et al., 2002). Future investigations should clarify how BHD modulates VEGF signaling to balance neurogenesis and vascular integrity, and employ long-term lineage tracing to confirm functional incorporation of migrating NPCs.

Notably, extracellular vesicles (EVs) derived from BHD-preconditioned Neural Stem Cells (NSCs) significantly accelerated neurological recovery in MCAO/R rats and enhanced NSCs proliferation/differentiation more effectively than BHD alone (Long et al., 2023). Beyond utilizing single-cell sequencing to investigate EV secretion mechanisms in NSCs stimulated by BHD’s active constituents, future efforts should focus on developing nano-delivery systems to efficiently deliver BHD’s holistic therapeutic profile rather than isolated components.

### 2.7 Promotion of angiogenesis

Reconstituting collateral blood flow *via* cerebral angiogenesis is vital for ischemic stroke recovery. In the infarcted region, angiogenesis drives microvascular sprouting and vascular remodeling—key steps in tissue repair (Ma et al., 2018). Over time, these new vessels deliver oxygen and nutrients to the neurovascular niche, fostering neuronal survival and regeneration (Arai et al., 2009).

BHD also targets Cav-1, potentially activating the Wnt signaling pathway and mediating effects through the metastasis-associated lung adenocarcinoma transcript 1 (MALAT1)/Yes-associated protein 1 (YAP1)/hypoxia-inducible factor 1α (HIF-1α) axis (Figure 6). This mechanism alleviates acute neurological deficits and pathological damage in MCAO/R mice, while promoting neural regeneration during recovery, increasing cortical blood flow and microvascular density in ischemic brain tissue (Chen et al., 2024; OuYang et al., 2025). The lncRNA MALAT1 is highly expressed in neural cells and participates in post-ischemic processes such as cell death, inflammation, and angiogenesis (Lipovich et al., 2012). Its neuroprotective and regulatory roles in pathological damage following cerebral ischemia have been confirmed in MCAO mouse models (Zhang et al., 2017). MALAT1 relies on Cav-1 for exosome internalization (Cooper et al., 2018; Wang et al., 2019). MALAT1 increases YAP1 nuclear translocation; YAP1 binds to and stabilizes HIF-1α protein, enhancing HIF-1α′s transcriptional activity to co-activate genes like VEGF, thereby promoting angiogenesis (Zhang X. et al., 2018; Sarkar et al., 2019; Liu et al., 2020). Functionally, this axis alleviates neurological deficits, enhances cortical perfusion, and increases microvascular density. Future studies should dissect the mechanisms of Cav-1–mediated exosome uptake and directly test MALAT1’s role in orchestrating YAP1/HIF-1α activity. It is worth noting that calycosin-7-O-β-D-glucoside from Radix Astragali may be a mediator through which BHD modulates Cav-1 (Fu et al., 2014).

Experimental evidence demonstrates that BHD upregulates VEGF and angiopoietin-1 (Ang-1), improving microvascular density (MVD). These pro-angiogenic effects are attenuated by Gap26, a connexin 43 (Cx43) inhibitor (Zhou et al., 2022). This indicates that Cx43 mediates BHD’s pro-angiogenic action *via* VEGF and Ang-1 (Figure 6). Cx43 is widely distributed in perivascular end-feet of astrocytes and vascular cells, providing structural and functional support for metabolic homeostasis within the neurovascular unit (McConnell et al., 2017; Bello et al., 2020). Studies have confirmed the pro-angiogenic role of Cx43 in endothelial cells, and phosphorylated Cx43 mediates the protective effects of erythropoietin on ischemic neurovascular unit injury (Koepple et al., 2021; Yu W. et al., 2021). Although the specific molecular interplay between Cx43 and VEGF/Ang-1 warrants further investigation.

Furthermore, BHD promotes post-stroke angiogenesis by targeting the SIRT1/VEGF signaling pathway (Figure 6) (Zheng et al., 2018; Tang et al., 2023). Tetramethylpyrazine, a component from *Ligusticum chuanxiong*, has been reported as a key active constituent potentially responsible for activating the SIRT1/VEGF pathway (Shu et al., 2024). SIRT1 binds the VEGF promoter to upregulate its transcription (Zhang H. et al., 2015). Upon secretion, VEGF engages VEGFR2 on endothelial cells to initiate pro-angiogenic signaling (Shibuya and Claesson-Welsh, 2006). Yet, VEGF also increases vascular permeability and edema by loosening endothelial junctions (Weis and Cheresh, 2005), posing a therapeutic paradox. Future studies should delineate how BHD balances VEGF’s angiogenic and permeability effects over acute and recovery phases, and identify the downstream mediators responsible for beneficial outcomes.

Mesenchymal stem cell (MSC) transplantation holds considerable promise for treating ischemic brain injury (Shen et al., 2012; Miyamoto et al., 2013). Studies show BHD-preconditioned MSCs secrete exosomes with elevated VEGF and miR-126—and reduced miR-221/miR-222—thereby upregulating VEGF and Ki-67 in recipient tissue and augmenting cerebrovascular density (Yang et al., 2015). Optimizing BHD’s modulation of MSC exosome cargo may enhance the clinical efficacy of MSC-based therapies.

### 2.8 Inhibition of excitotoxicity

Mitigating excitotoxicity is an essential strategy for treating ischemic stroke (Chamorro et al., 2016). After ischemia, ATP depletion causes membrane depolarization and calcium overload. Simultaneously, excessive release of glutamate (GLU) and aspartate (ASP) from presynaptic terminals overstimulates NMDA and AMPA receptors, allowing massive Ca^2+^ and Na^+^ influx. This ionic imbalance drives ROS production, lipid peroxidation, and cytoskeletal breakdown, culminating in neuronal death (Belov Kirdajova et al., 2020; Choi, 2020; Baranovicova et al., 2023).

A study by Wang L et al. demonstrated that a 7-day intervention with BHD reduced elevated levels of glutamate (GLU) and aspartate (ASP) in the cerebrospinal fluid (CSF) of MCAO/R model rats, while increasing the levels of inhibitory amino acids—glycine (Gly), taurine (Tau), and γ-aminobutyric acid (GABA) (Wang et al., 2013). Nonetheless, how BHD modulates brain amino acid pools is unknown. Since the glutamate–glutamine cycle in astrocytes critically maintains excitatory–inhibitory balance and supports neuronal viability during ischemia (Stelmashook et al., 2011), future work should test whether BHD acts by enhancing astrocytic glutamine synthetase or glutamate uptake.

Glutamate transporter-1 (GLT-1) mediates over 90% of synaptic glutamate uptake into astrocytes for conversion to glutamine by glutamine synthetase (GS) (Zou et al., 2010; Krzyżanowska et al., 2014). During ischemia, GLT-1 and GS are downregulated, worsening excitotoxicity (Krzyżanowska et al., 2014). BHD was shown to increase the level of pituitary adenylate cyclase-activating polypeptide 38 (PACAP38) in the subacute phase of MCAO/R model rats. PACAP38 promotes the upregulation of GLT-1 and GS expression in the hippocampal region—an effect that can be blocked by a PACAP38 inhibitor (Ding et al., 2015). However, the study did not assess resulting changes in infarct size or neurological outcomes. Moreover, as GLT-1 is astrocyte-specific, it remains to be determined whether BHD’s action is directly astrocytic or mediated *via* other cell types.

Glutamate not only mediates fast synaptic transmission *via* ionotropic receptors (iGluRs) but also activates metabotropic receptors (mGluRs) that modulate intracellular signaling (Bodzęta et al., 2021). In ischemia, mGluR1 signaling worsens neuronal injury (Yawata et al., 2008), highlighting glutamate receptor modulation as an anti-excitotoxic strategy (Shen et al., 2022). Research by Zhao L et al. confirmed that BHD downregulated both the mRNA expression of mGluR1 and glutamate levels in the striatum during the acute phase of cerebral I/R model rats. This was accompanied by improved behavioral scores and reduced cerebral infarct volume 3 days after I/R (Zhao et al., 2012). However, the pathways by which BHD decreases glutamate release and mGluR1 expression—and whether it selectively targets specific receptor subtypes—remain unknown.

### 2.9 Regulation of material and energy metabolism

Proper energy metabolism is essential for neuronal survival. After ischemic stroke, reduced perfusion and tissue damage disrupt metabolic homeostasis, instigating calcium overload, neuroinflammation, mitochondrial failure, and excitotoxic cascades (Zhou et al., 2021; Awasthi et al., 2024). Thus, restoring metabolic balance is a key therapeutic goal (Villa et al., 2013).

Studies indicate that BHD modulates post-ischemic energy metabolism disturbances through multiple mechanisms. On one hand, BHD has been shown to upregulate the expression of glucose transporters (GLUTs) and monocarboxylate transporters (MCTs) in the ischemic cortex of MCAO/R rats during the recovery phase (Li M. et al., 2024), suggesting its potential to enhance glucose and lactate transport. However, further quantification of actual metabolic flux changes using techniques such as isotopic tracing is still required. Moderate glycolysis during hypoxia maintains glial and neuronal viability, and the resulting lactate can drive angiogenesis (Bouzat and Oddo, 2014; Zeng et al., 2021; Dong et al., 2022). Moreover, Tian F report that BHD activates AMPK in ischemic brain, suggesting a role in sustaining glycolytic metabolism and perfusion (Tian et al., 2024). Moreover, based on preliminary evidence from metabolomics and functional validation, BHD may correct post-ischemic cerebral energy metabolism dysfunction by modulating the SIRT1/AMPK axis to promote glucose uptake, activate glycolysis and the tricarboxylic acid (TCA) cycle, and restore mitochondrial respiratory function (Hu et al., 2025). Confirming AMPK’s direct involvement will require targeted AMPK inhibition studies.

Regarding neurometabolic balance, Wang R further link BHD’s neuroprotection to sphingolipid and inositol phosphate metabolism (Wang et al., 2024). Together, untargeted metabolomics (Tang et al., 2022) and multi-omics analyses (Zhou et al., 2023) converge on altered purine, glycerophospholipid, glycosphingolipid, and glutamate pathways in the ischemic hippocampus. Notably, post-IS glutamate accumulation triggers delayed neuronal degeneration and death cascades (Krzyżanowska et al., 2014; Lai et al., 2014). However, key enzymes and transporters mediating these shifts remain unvalidated. To translate these findings, future work should pair proteomic target confirmation with analysis of human stroke specimens to establish robust metabolic biomarkers of BHD efficacy.

### 2.10 Regulation of gut microbiota

Alterations in gut microbiota composition strongly influence ischemic stroke pathophysiology and recovery (Zhang et al., 2023). Evidence suggests gut dysbiosis plays a critical role in IS (Peh et al., 2022), primarily mediated *via* the gut-brain axis through pro-inflammatory immune responses and the accumulation of microbial metabolites (Singh et al., 2016). Notable metabolites include short-chain fatty acids (SCFAs), trimethylamine N-oxide (TMAO), tryptophan catabolites, and bile acids (BAs) (Peng et al., 2018; Peng et al., 2018).

In humans, IS patients exhibit reduced gut microbiota diversity with increased abundance of *Actinobacteria*, *Proteobacteria*, Bacteroidaceae, and Bifidobacteriaceae, alongside decreased *Bacteroidetes*, *Firmicutes*, *Eubacterium*, *Faecalibacterium*, and *Roseburia* (Peh et al., 2022). Reduced SCFA levels, particularly acetate, correlate with poor 3-month outcomes in a case-control study of 140 acute IS (AIS) patients (Tan et al., 2021). In rodent models, stroke disrupts gut physiology—slowing motility and promoting bacterial overgrowth (Durgan et al., 2019). Transplanting dysbiotic microbiota from stroke donors into germ-free mice increases infarct size and neurological deficits upon MCAO (Singh et al., 2016; Xia et al., 2019).

Targeting Enterobacteriaceae in MCAO mice reduces systemic inflammation and hippocampal injury, whereas higher *Lactobacillus* levels associate with reduced apoptosis and smaller infarcts in stroke rats (Wanchao et al., 2018; Xu et al., 2021). BHD similarly enriches beneficial taxa (e.g., *Lactobacillus*) and suppresses pathogenic genera (e.g., Escherichia–Shigella, *Klebsiella*) in the MCAO gut microbiome. These alterations may modulate hippocampal metabolism (Tang et al., 2022), yet the causal chain linking microbial shifts and neuroprotection remains to be firmly established.

Recent investigations on individual active constituents of BHD have provided more direct experimental evidence for the proposed causal links. Calycosin has been reported to modulate gut microbiota and bile acid metabolism, thereby activating intestinal FXR signaling, which in turn upregulates tight junction proteins (ZO-1, Occludin) in both the colon and brain, ultimately attenuating neuroinflammatory injury in cerebral ischemia-reperfusion models (Zhou et al., 2025). Similarly, astragaloside IV, despite its low oral bioavailability, has been shown in several animal studies to exert protective effects by reshaping gut microbiota composition, restoring intestinal barrier integrity (reducing plasma LPS leakage), and regulating serum metabolic profiles, particularly amino acid metabolism and the PPAR signaling pathway. In addition, astragaloside IV can activate the Nrf2 antioxidant pathway, thereby maintaining tight junction proteins in brain microvascular endothelial cells and mitigating blood–brain barrier disruption (Li et al., 2018; Li Z. et al., 2023; Xu et al., 2018).

Collectively, these findings suggest that BHD and its constituents may act through a multilayered network: initially by modulating gut microbiota, subsequently altering microbial metabolites and systemic endotoxin burden, and ultimately strengthening intestinal and blood–brain barriers while suppressing systemic and central inflammation to facilitate brain tissue repair. It should be emphasized, however, that most of the current evidence is derived from animal studies or single-compound interventions, and is insufficient to establish a complete causal chain in the context of the whole formula. To substantiate the pathway of “BHD → gut microbiota/metabolite modulation → barrier restoration → neuroprotection,” future studies should employ formula-level causal experiments (e.g., fecal microbiota transplantation, germ-free animal models, supplementation or inhibition of key strains/metabolites, barrier function assays), and further compare the interactions and potential synergy between isolated compounds and the full decoction.

## 3 Conclusion and perspectives

BHD is a classical TCM formula for ischemic stroke that embodies the principles of “multi-component, multi-target, and holistic regulation.” Clinical reports and preclinical studies suggest that BHD can improve neurological outcomes and functional recovery with a generally acceptable safety profile (Shao et al., 2022; Wang et al., 2022). However, high-quality, large-scale randomized trials remain limited. Mechanistic work to date indicates that BHD exerts synergistic neuroprotective effects across multiple biological processes, including attenuation of neuroinflammation and oxidative stress, modulation of apoptosis and autophagy, promotion of neurovascular repair, reprogramming of cerebral energy metabolism, and regulation of gut microbiota composition.

A key finding that emerges from this systematic review is the multifunctional role of several core signaling pathways—such as PI3K/Akt, SIRT1, and AMPK—in mediating the pleiotropic effects of BHD. Rather than acting in isolation, these pathways form a complex, interconnected network that is dynamically engaged across different pathological contexts. For instance, the PI3K/Akt axis is recruited to suppress neuroinflammation, inhibit neuronal apoptosis, and promote angiogenesis. Similarly, SIRT1 activation contributes to the regulation of autophagy, energy metabolism, and vascular repair. This context-dependent multiplexing of core pathways underscores a fundamental advantage of polypharmacological agents like BHD: the ability to synchronously modulate multiple disease-relevant processes through a limited set of highly leveraged signaling hubs. Future research should prioritize mapping the cross-talk between these hubs and delineating how their engagement varies by cell type and temporal phase after stroke.

Importantly, available evidence supports a stage-dependent view of BHD’s actions that aligns with the evolving pathology after cerebral ischemia. In the acute phase, BHD primarily exerts neuroprotective effects by swiftly countering the initial damage cascade. This is achieved through robustly inhibiting neuroinflammation (e.g., *via* suppressing NLRP3 inflammasome), alleviating oxidative stress (e.g., *via* activating the Nrf2 antioxidant pathway), and reducing excitotoxicity and apoptosis, thereby stabilizing the ischemic penumbra and limiting infarct expansion.During the subacute and recovery phases, BHD’s role strategically shifts from protection to reconstruction and repair. Its mechanisms pivot towards promoting neurovascular remodeling (e.g., *via* enhancing angiogenesis through VEGF signaling and synaptogenesis *via* CREB activation), regulating metabolic reprogramming (e.g., *via* SIRT1/AMPK axis), and restoring systemic homeostasis (e.g., *via* modulating peripheral immunity and gut microbiota). This multi-faceted approach underpins its efficacy in facilitating long-term neurological and functional recovery. These stage-specific patterns are supported mainly by animal and *in vitro* data; translation to defined clinical time windows requires further validation. Current evidence suggests that BHD’s therapeutic effects likely arise from the synergy among: (1) direct actions of brain-penetrant compounds on neuronal and glial targets; (2) peripheral immunomodulation that mitigates systemic inflammation and secondary brain injury; and (3) remodeling of the gut microbiome and production of neuroactive metabolites that influence brain function *via* the gut-brain axis. This multi-pathway model aligns well with the holistic philosophy of TCM and helps explain how BHD can coordinate restorative responses across multiple organ systems.

Several critical gaps must be addressed to advance BHD toward evidence-based, precision use. First, mechanistic studies have largely traced isolated signaling nodes; the crosstalk among pathways, the cell-type specificity of effects (neurons *versus* microglia, astrocytes, endothelial cells, *etc.*), and the temporal dynamics across defined post-ischemic windows remain incompletely characterized. Second, although multiple bioactive constituents (for example, astragaloside IV and paeoniflorin) have been identified (Liu et al., 2022), the net therapeutic effect likely arises from complex interactions (synergy, additivity, or antagonism) among many compounds; rigorous dissection of these interactions is lacking. Third, practical translational challenges—bioavailability, brain delivery, formulation standardization, and optimized dosing/time-window—require targeted solutions.

To address these gaps we recommend a coordinated, hypothesis-driven research agenda combining mechanistic precision and translational relevance. Key experimental approaches should include: (1) targeted pharmacokinetics and BBB penetration studies using labeled compounds to quantify brain exposure and metabolite formation; (2) cell-type-specific interventions, such as conditional (cell-specific) knockouts or genetic fate-tracing, to determine which cell populations mediate particular effects; (3) single-cell and spatial omics across multiple post-ischemic time points to resolve spatiotemporal pathway activation; (4) metabolic flux analyses (stable isotope tracing) to quantify changes in glucose/lactate/TCA flux and link transporter expression to functional metabolism; (5) gut-brain causal experiments, including germ-free models and fecal microbiota transplantation, to test whether microbiota shifts mediate neuroprotection; and (6) combinatorial pharmacology (fractionation, reconstitution, and systems pharmacology) to map synergy/antagonism among constituent groups. Parallel development of brain-targeted delivery platforms (e.g., nanoparticle or exosome carriers) should be pursued to improve CNS bioavailability where appropriate.

In summary, BHD represents a promising multi-target therapeutic strategy for ischemic stroke whose biological rationale is increasingly supported by preclinical data. Realizing its translational potential will depend on combining modern mechanistic tools with rigorous pharmacology and carefully timed clinical studies to define which components act where and when—and thereby to optimize formulations, delivery, and patient selection.
