# Supplementary material for: Neuroprotective mechanisms of Buyang Huanwu decoction in ischemic stroke
Source: Front Pharmacol. 2025 Sep 23;16:1620533. doi: 10.3389/fphar.2025.1620533 (PMC12500392; doi:10.3389/fphar.2025.1620533)
Supplement: Supplementary file 2 [file DataSheet1.docx]

**Literature Search Strategy**

**1. Search Databases**

To identify relevant studies, systematic searches were conducted in the following electronic databases:

- **PubMed**: A leading database covering international biomedical literature.
- **Web of Science Core Collection**: Including **Science Citation Index Expanded (SCIE)** (core journals in natural sciences), Social Sciences Citation Index (SSCI) (core journals in social sciences), and Emerging Sources Citation Index (ESCI).
- **China National Knowledge Infrastructure (CNKI)**: Containing **Chinese core journals (Peking University Core)** and Chinese Science Citation Database (CSCD) source journals.

*Rationale for Selection*:

- The **Web of Science Core Collection** screens journals through 28 rigorous quality criteria and impact assessments, ensuring inclusion of the most influential SCI journals globally across disciplines.
- CNKI’s "Core Journal Navigation" and "Source Category" filtering functions enable precise targeting of Chinese core journals, covering authoritative research in traditional Chinese medicine.

**2. Search Terms and Search Strings**

Boolean operators (*AND*, *OR*) were used to combine **core concepts** into search strings, adjusted to each database’s syntax:

- **Term Set 1 (Buyang Huanwu Decoction)**: "Buyang Huanwu Decoction", "BHD", "Buyang Huanwu Tang", "Bu Yang Huan Wu Decoction" (including common romanization variants).
- **Term Set 2 (Ischemic Conditions)**: "ischemic stroke", "cerebral ischemia", "cerebral ischemia reperfusion", "cerebral I/R injury", "ischemic cerebrovascular disease".

**Example Search Strings**:

- **Web of Science**:

TS=("Buyang Huanwu Decoction" OR "BHD" OR "Buyang Huanwu Tang") AND TS=("ischemic stroke" OR "cerebral ischemia reperfusion" OR "cerebral I/R injury")

Databases=SCI-EXPANDED, SSCI, ESCI // Limited to SCIE, SSCI, and ESCI databases

- **CNKI**:

Subject=("补阳还五汤" OR "BHD") AND Subject=("缺血性脑卒中" OR "脑缺血再灌注")

Source Category=Core Journals // Filter by "Core Journals" category

**3. Timeframe and Languages**

- **Publication Period**: Articles published between January 2000 and April 2025 were included.
- **Languages**: Only studies published in English or Chinese were included to ensure accessibility for full-text analysis.

**4. Inclusion Criteria**

- **Study Type**: Original research (including in vivo/in vitro experiments, clinical trials, and observational studies).

*Note*: This scope covers major types of original research from basic to clinical studies, aligning with common study designs in ischemic brain injury and traditional Chinese medicine mechanism research.

- **Interventions**: Studies investigating the complete Buyang Huanwu Decoction formula or formulations with only minor modifications.
- **Journal Sources**:
  - **International Studies**: Published in journals indexed in **SCIE, SSCI, or ESCI** (filtered via Web of Science Core Collection).
  - **Domestic Studies**: Published in **Chinese core journals (Peking University Core)** or Chinese Science Citation Database (CSCD) source journals (filtered via CNKI).
- **Study Outcomes**: Research focusing on the mechanisms of action of Buyang Huanwu Decoction in ischemic stroke or cerebral ischemia-reperfusion injury.

**5. Exclusion Criteria**

- Studies using partial or highly simplified versions of Buyang Huanwu Decoction (e.g., extracts of single herbs from the formula rather than the complete decoction).
- Non-original research: Reviews, conference abstracts, editorials, news articles, or animal studies unrelated to cerebral ischemia models.
- Studies with incomplete data (e.g., unavailable full text, unclear methodology, or irrelevant outcome measures).
- **Ineligible journal sources**: Literature not indexed in SCIE, SSCI, ESCI, Chinese core journals, or CSCD.

**6. Search Process**

1. **Database Searches**:
   - **Web of Science**: Searched via the "Core Collection", limited to SCIE, SSCI, and ESCI databases, using the above search string.
   - **CNKI**: Searched using the "Advanced Search" function, with the "Core Journals" source category selected, applying the Chinese search string.
   - **PubMed**: Searched using the English search string; subsequent verification via Web of Science confirmed whether journals were indexed in SCIE.
2. **Literature Management**: Citations were merged using Zotero software, with duplicate records automatically removed.
3. **Preliminary Screening**: Two independent reviewers excluded obviously irrelevant studies (e.g., non-original research, ineligible journal sources) based on titles and abstracts.
4. **Full-Text Evaluation**:
   - **International Journals**: Verified via the Web of Science "Journal List" to confirm indexing in SCIE, SSCI, or ESCI.
   - **Chinese Journals**: Confirmed as Chinese core journals or CSCD source journals via CNKI’s "Journal Navigation" or "Source Category" labels.
5. **Dispute Resolution**: Disagreements were resolved through consensus or consultation with a third reviewer.

This strategy ensures that included literature is derived from Chinese core journals or SCI-indexed journals through dual mechanisms of **database limitations** (e.g., Web of Science’s SCIE filter, CNKI’s core journal screening) and **manual verification** (journal list checks), significantly enhancing the academic quality and credibility of the research.
